# Social Media Depictions of the Impact of Noise Pollution on Communication and Mental and Physical Health

**DOI:** 10.1007/s10900-025-01457-7

**Published:** 2025-03-13

**Authors:** Betty Kollia, Corey H. Basch, Eunsun Park, Helen Yousaf

**Affiliations:** 1 Department of Speech-Language Pathology, William Paterson University, Wayne, NJ 07470 USA; 2 Department of Public Health, William Paterson University, Wayne, NJ 07470 USA

**Keywords:** Noise pollution, YouTube, Noise induced hearing loss, Community well-being, Communication and cognition, Stress

## Abstract

Noise pollution is known to have harmful consequences on various facets of human health, as is recognized by the United States Centers for Disease Control and Prevention as well as by the World Health Organization. Even though noise pollution is a ubiquitous and global problem, the public may not be cognizant of its ill-effects. Often, people go online first to obtain information, and YouTube is the second most used social media platform. For these reasons, the objective of this study was to examine the information available by YouTube to the public about parameters pertaining to noise pollution. The most viewed one-hundred videos in English were obtained for examination from YouTube, searching for “*noise pollution.”* Each video was assessed regarding the pertinent information (content) it displayed, as well as for the date it was uploaded, the source of its upload (professional or consumer/public), the duration of the video (in seconds), number of times it was viewed, and the number of “likes” it received. Descriptive statistics, Mann-Whitney U tests, Fisher’s exact tests, or Chi-square tests were applied as appropriate. Analysis of video characteristics indicated that the 100 most viewed videos on YouTube on the topic of noise pollution were uploaded from 2009 to 2023, with the highest frequency of uploads occurring in 2022 (16%), with 27% originating from the general public and 73% originating from professional health organizations. Cumulatively, the videos were viewed over 15 million times, and those uploaded by consumers had a greater median view tally (17,000), were longer in duration, and were “liked” more compared to videos uploaded by professionals (5,100). However, these differences were not statistically significant. Analysis of video content provided a breadth of data, with statistically significant differences (*p* < 0.05) between consumer and professional videos in their presentations of noise characteristics, discussions of official and anecdotal complaints regarding noise, health problems attributed to noise pollution, general effects of noise on the community, and reactions from community administrators. Further topics shown equally in consumer and professional videos, from tinnitus to communication difficulties are discussed. Whereas of the one hundred most viewed videos, 73 were uploaded by professionals compared to 27 uploaded by the public, the fact that the professional videos had a somewhat lower median number of views (5,100 compared to 17,000), may point to a likely preference of consumers for videos by “peers.” It may be worthwhile for professional organizations to consider this tendency so as to enhance the public’s accessing of curated professional videos. Concerning the content available, the findings reveal that there is a respectable professional presence on YouTube videos on noise pollution. Nonetheless, certain issues were not adequately addressed, including the significant cognitive, communication, and physical harmful effects, or possible information that could be useful to the public on noise exposure.

## Introduction

The term “noise pollution” is associated with unwanted and harmful noise exposure that can be frequent and excessive  [1, 2]. Noise pollution has an injurious impact on one’s physical (e.g., tinnitus, hearing loss, cardiovascular disease, etc.) and mental health (e.g., stress, decreased cognitive function, memory and attention problems, etc.) [3]. The United States Centers for Disease Control and Prevention (CDC) underscore that persistent loud noise can cause irreversible sensorineural hearing loss by indelibly damaging the cilia in the inner ear, which are responsible for sending auditory signals to the brain [4]. According to the Occupational Safety and Health Administration (OSHA) and the CDC, Noise-Induced Hearing Loss (NIHL) can be permanent, bilateral, and can have a profound impact on a person’s ability to communicate, because it severely reduces hearing in the speech and higher frequencies, which impairs comprehension of speech [Occupational Safety and Health Association, [5, 6]. Various professional health organizations have consistently noted the ill effects of loud noise exposure on human health. The American Speech-Language Hearing Association (ASHA) provides similar best practices advice as the CDC and informs visitors to its website (ASHA.org) that NIHL is dependent not just on how loud the noise is, but also on the proximity of the individual to the noise source and on the length of the person’s exposure to the loud noise (American Speech-Language Hearing Association, [7]). Quantifying exposure is an important aspect of addressing noise pollution concerns, as the CDC states, but currently advanced modeling techniques are lacking [8]. Researchers acknowledge the need for increased funding, research, and data to improve our understanding of noise influences on health outcomes [9]. A recent meta-analysis of epidemiologic studies on the detrimental effects of noise on cognitive skills, revealed that children’s language and reading comprehension and middle-aged adults’ cognitive functions were negatively affected by environmental noise [10]. Along with hearing loss, chronic and loud noise exposure increases cortisol and adrenaline levels, causing oxidative stress that may precipitate anxiety and stress and, thereby, result in high blood pressure and heart disease [11]. Moreover, high-intensity sound levels—85 decibels or higher—are known to have harmful physical and psychological consequences  [12]. Noise pollution at such high levels has been linked to short-term deleterious health effects, including stress and sleep disturbances [13]. Recognized long-term adverse health outcomes include cardiovascular disease, neurological damage, and hearing impairment  [14, 15]. Environmental noise contributes to premature deaths and ischemic heart disease [16, 17]. Further, the effects of noise on the brain and nervous system are widespread and include the impairment of cognitive function, oxidative stress, depression, anxiety, and neurodegenerative disorders [11–19].

The CDC and the World Health Organization (WHO) recommend protection strategies at the individual, community, federal, and global levels to ensure that loud noise exposure is reduced [20, 21]. The federal government’s role in mitigating noise pollution involves increasing public awareness of the negative impacts of loud noise exposure, creating, and updating appropriate standards, monitoring noise emissions, and promoting research on hearing loss in the US, as outlined in the Healthy People 2020 objectives [22].

Despite its pervasive prevalence globally, the public may be unaware of the pernicious effects of noise pollution [23, 24]. Many people seek information first online, and specifically on YouTube [25]. YouTube has several million new and unique viewers. In fact, as of January this year, YouTube is the second most used social media app with 2,491 users (in millions), preceded only by Facebook. Therefore, the information available on this platform about noise pollution merits examination [26].

## Materials and Methods

The study’s methodology followed previously established guidelines [27]. One-hundred English-language videos were extracted for analysis from YouTube, after searching with the term “*noise pollution.”* Results were filtered by “view count.” Each video was examined for its content regarding the noise information it presented as well as for the date of its upload, source (professional or consumer/public), video duration (in seconds), number of views, and number of likes. The number of views and likes are considered indices of the video’s reach and potential impact on the public.

The video content categories were examined in relation to:

- characteristics of noise: source and proximity to source, number of hours of noise, and intensity of noise (in dB);
- problems associated with noise: official and anecdotal complaints, health problems, quality of life, offenders and solutions;
- response: impact on community, administrative response, health provider recommendations,
- mention of research information.

All content categories were coded as present or not present.

Descriptive statistics were used to explore the characteristics of noise pollution content in YouTube videos. We employed the Mann-Whitney U test to examine differences in views, likes, and video duration between videos uploaded by consumers and by professionals. This test is specifically designed to assess the distributions of two independent samples, effectively accommodating for any differences in sample size between the groups [28]. The Chi-square test or Fisher’s exact test were used to compare the prevalence of content topics within videos from these two sources. All statistical analyses were conducted utilizing IBM SPSS Statistics software for Windows (version 28, Armonk, NY). A value of *p* < 0.05 was used to determine the statistical significance of the results.

## Results

### Video Characteristics

**Fig. 1:**
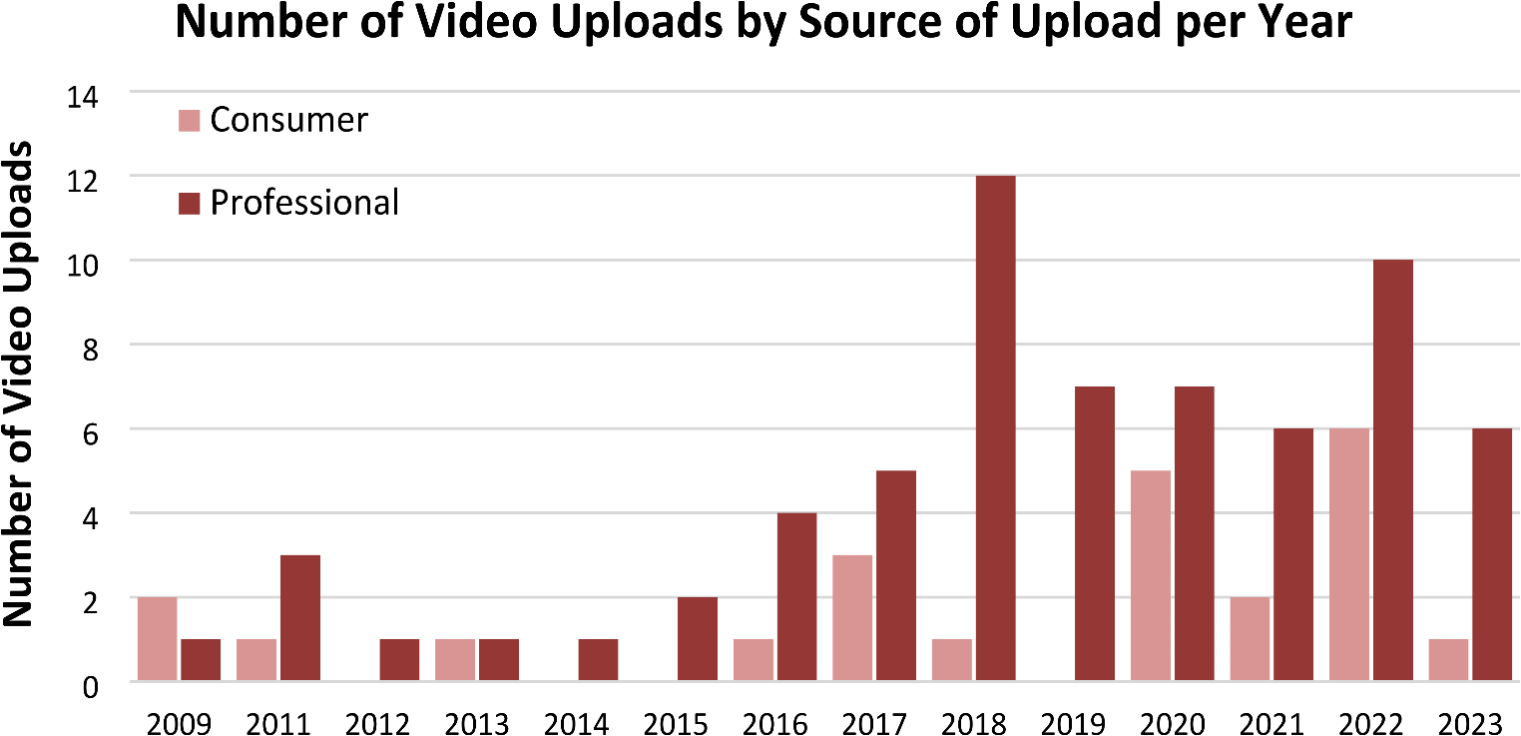
Number of video uploads by source of upload (consumer vs. professional), per year, for the period 2009–2023 for the 100 most viewed YouTube videos on Noise Pollution

- *Period of upload*: The 100 most viewed videos on YouTube on the topic of noise pollution were uploaded from 2009 to 2023, with the highest frequency of uploads occurring in 2022 (16%), followed by 2018 (13%), 2020 (12%), and 2017 (11%) (Fig. 1).
- *Source of upload*: The videos were categorized based on the uploader’s profile, with 27% (n = 27) originating from “consumers” (general public) and 73% (n = 73) originating from “professionals” (professional health organizations).
- *View Counts*: The cumulative view count for the videos reached 15,193,355. For each video, the view counts ranged from 266 to 3,900,000 views. Because this range was not normally distributed, the median, rather than the mean, was selected as a more representative index of the central tendency for the number of “view counts,” “likes,” and duration of the videos. The median of “view counts” for the videos was 9,050. Consumer-uploaded videos had a higher median view count of 17,000 compared to 5,100 for professional uploads.
- *“Likes:”* The overall median number of “likes” per video was 64.00 (range: 2–93,000). Consumer videos had a higher median of likes at 144, as opposed to a median of 47 for professional videos.
- *Duration of videos*: The median video duration was 176.00 sec (range: 37 − 1,555 sec). Consumer videos were longer, with a median duration of 239 sec, compared to 164 sec for professional videos.

While the medians in the numbers of the video characteristics noted above were different, the Mann-Whitney U test showed no statistically significant differences between the consumer and professional videos in terms of view counts (*p* = 0.07), number of likes (*p* = 0.16), and video durations (*p* = 0.23) (see Table 1).

**Table 1:** Noise pollution YouTube video characteristics: total, by source of upload (Consumer and Professional), and comparison of differences in median number of views, “likes,” and duration of videos

Characteristic	Total (*n* = 100)	Consumer (*n* = 27)	Professional (*n* = 73)	*p*
Views (x1000) Range [min, max]	9.05 [0.3, 3,900.0]	17.00 [0.7, 1,800.0]	5.10 [0.3, 3,900.0]	0.08
Likes Range [min, max]	64.00 [2, 93,000]	144.00 [2, 34,000]	47.00 [4, 93,000]	0.22
Length (seconds) Range [min, max]	176.00 [37, 1,555]	239.00 [72, 1,408]	164.00 [37, 1,555]	0.35

*p*-values derived from Mann-Whitney U Test. No comparisons reached statistical significance (0.05)

### Content Analysis

Analysis of the content presented in the videos revealed a diverse range of information. Differences in the content coverage by source of upload were examined via the Chi-square test or Fisher’s exact test. Four of the comparisons yielded statistically significant differences (*p* < 0.05), as noted below.

- *Characteristics of noise*: A total of 19 videos discussed decibel levels associated with noise pollution, 88 videos addressed the various sources of noise, and 12 videos addressed specific times when noise was prevalent.
- *Problems associated with noise*: Seven videos referenced official complaints regarding noise pollution, whereas 86 videos included anecdotal accounts of noise-related complaints. A statistically significant disparity was observed in the discussion of noise-related complaints between consumer (*n* = 2) and professional (*n* = 21) uploads (*p* = 0.03).
  Discussions regarding health issues related to noise pollution appeared in 36 videos with a significant difference between consumer (*n* = 5) and professional (*n* = 31) uploads (*p* = 0.02).
  Moreover, 35 videos noted the impact of noise pollution on quality of life, eight videos identified alleged noise pollution offenders, and 46 videos proposed solutions to mitigate noise pollution.

c. *Response*: Notably, the general effects of noise on the community were addressed in 21 videos, with a significant difference between consumer (*n* = 2) and professional (*n* = 19) (*p* = 0.05). Responses from administrative entities or local authorities were featured in 27 videos, with a significant variation in coverage between consumer (*n* = 3) and professional (*n* = 24) uploads (*p* = 0.02). Recommendations from healthcare providers were seen in only a few videos (*n* = 10), as was inter-professional communication regarding noise pollution (*n* = 7), with no statistically significant differences between sources of upload.
d. *Mention of research information*: references to research findings were seen in one fifth of the videos (*n* = 20) at a similar rate among the two sources of upload.

Additional topics appearing in the videos, though without statistically significant differences between consumer and professional uploads, included tinnitus/ringing in the ears (*n* = 7), specific locations affected by noise pollution (*n* = 10), the impact of noise pollution on animals (*n* = 23), and other behavioral and physical health issues linked to noise exposure, such as difficulties in communication, increased irritability, sleep disturbances, high blood pressure, diabetes, obesity, heart disease, and instances of death (see Table 2).

**Table 2:** Topics regarding noise pollution addressed in YouTube videos uploaded by consumers and professional sources

Source of Upload Topics addressed in the video	Total (*n* = 100)	Consumer (*n* = 27)	Professional (*n* = 73)	*p*
Noise-related dB levels	19	3	16	0.26
Specific noise hours	12	3	9	1.00
Specific noise sources	86	24	62	1.00
Official complaints related to noise	8	1	7	0.44
Anecdotal complaints related to noise	23	2	21	0.03*
Health complaints related to noise	36	5	31	0.02*
Quality of life impacted by noise	35	6	29	0.10
Solutions offered	46	12	34	0.83
“Offenders” named	8	1	7	0.44
General effects/impact of noise on the community	21	2	19	0.05*
Administrative response to noise	27	3	24	0.02*
Recommendations from health providers regarding noise pollution	10	1	9	0.28
Research findings mentioned about noise pollution	20	5	15	1.00
Specific geographic locations noted (e.g. proximity to airports, subways, etc.)	11	2	9	0.72
Impact of noise on interpersonal communication	7	0	7	0.19
Impact on animals	23	6	17	0.75
Tinnitus/ringing in the ears	7	2	5	0.82
Having to talk louder	0	0	0	1.00
Feeling of missing out on conversation due to noise	2	0	2	0.55
Avoiding or finding unpleasant communication with other people due to noise	6	1	5	0.67
Having to turn up the volume on TV or other media	2	0	2	0.55
Having difficulty focusing/concentrating on work, reading, study	19	4	15	0.40
Feeling irritable/annoyed due to the noise	46	10	36	0.34
Headaches	4	1	3	0.54
Sleep disturbances	35	6	29	0.16
High blood pressure	16	2	14	0.26
Diabetes	4	0	4	0.29
Obesity	3	0	3	0.36
Heart disease	23	4	19	0.35
Death	5	1	4	0.76

*p*-values derived from chi-square test for independence or Fisher’s exact test, when warranted by insufficient cell counts

* indicates statistically significant value

## Discussion

Even though the videos uploaded by professionals were almost three times as numerous as videos uploaded by consumers (73 compared to 27), professional videos had a somewhat lower median number of views (5,100 compared to 17,000) than did consumer videos. While this difference was not statistically significant, it shows a possible preference of the public toward peer videos rather than professional ones. Professional organizations may wish to examine the possible reasons for this trend and improve traffic to professional videos.

Regarding content coverage, the findings of this study suggest that, despite a reasonable professional presence in the most widely viewed YouTube videos on noise pollution, the majority of videos did not sufficiently address the considerable mental, cognitive, and physical adverse health effects, or offer potential answers that could be useful to the public on noise exposure. A limitation of this study is the small, cross-sectional sample. However, it provides insight into a topic impacting public health, on which little research exists. Recent research suggests that YouTube users seek health-related content on that platform and are often guided by its information [29]. Therefore, more comprehensive and interprofessional content on noise pollution and health would benefit those who seek information for overall education, prevention, and treatment.

A 2022 WHO report offered guidance on mitigating noise pollution’s hazardous impacts at the local and national levels [20]. These hazards are not trivial. The 100 video uploads analyzed herein indicate that excessive noise continues to be a danger to both human and animal life; however, of these, only 16 discuss the deleterious health effects of noise on people and animals, while none of the videos offer specific recommendations from healthcare professionals regarding prevention or treatment. The American Academy of Audiology has a wealth of online information for specialists and consumers, including briefs on recent pharmacological advances for preventing NIHL (in a mouse model) [30], or the ways in which noise impacts animals  [31], and this is useful material. When it comes to hearing conservation, prevention is paramount, and mentions of scientific data were insufficient in the videos we analyzed.

The effect of noise on quality of life was noted in about one third (37) of the videos, while noise effects on interpersonal communication were mentioned in only two videos. To increase the public’s health literacy levels on the topic of noise-induced hearing loss and noise pollution, a more impactful professional presence and increased content specificity are required, and social media such as YouTube, provide a popular forum that can be better utilized for this purpose.

## Data Availability

Data will be available upon reasonable request.

## References

[1] Benisek, A. (2024, Feburary 20). What is noise pollution? WebMD. https://www.webmd.com/a-to-z-guides/what-is-noise-pollution

[2] Nathanson, J. A., Berg, R. E., Beeson, R., Augustyn, A., Curley, R., Gaur, A., Gregersen, E., Bhutia, K. T., Lotha, G., Petruzzello, M., & Tikkanen, A. (2024). April 30). Noise pollution. Britannica. https://www.britannica.com/science/noise-pollution

[3] Dutchen, S. (2022). Noise and Health. Harvard Medicine. https://magazine.hms.harvard.edu/articles/noise-and-health

[4] Centers for Disease Control and Prevention (2024d, April 12). Preventing Noise-Induced Hearing Loss. https://www.cdc.gov/nceh/hearing_loss/how_do_i_prevent_hearing_loss.html

[5] Occupational Safety and Health Association (n.d.). Occupational noise exposure. United States Department of Labor. https://www.osha.gov/noise/health-effects

[6] Centers for Disease Control and Prevention (2024b, February 16). Understand noise exposure. https://www.cdc.gov/niosh/topics/noise/preventoccunoise/understand.html

[7] American Speech-Language Hearing Association (n.d.) Loud noise dangers. https://www.asha.org/public/hearing/loud-noise-dangers/

[8] Centers for Disease Control and Prevention (2024c, May 15). Types of hearing loss. https://www.cdc.gov/ncbddd/hearingloss/types.html

[9] Powell, D. S., Oh, E. S., Reed, N. S., Lin, F. R., & Deal, J. A. (2022). Hearing loss and cognition: What we know and where we need to go. *Frontiers in Aging Neuroscience*, *13*, 769405. 10.3389/fnagi.2021.769405 35295208 10.3389/fnagi.2021.769405 PMC8920093

[10] Thompson, R., Smith, R. B., Karim, B., Shen, Y., Drummond, C., Teng, K., C., & Toledano, M. B. (2022). Noise pollution and human cognition: An updated systematic review and meta-analysis of recent evidence. *Environment International*, *158*, 106905. 10.1016/j.envint.2021.106905 34649047 10.1016/j.envint.2021.106905

[11] Hahad, O., Prochaska, J. H., Daiber, A., & Muenzel, T. (2019). Environmental Noise-Induced effects on stress hormones, oxidative stress, and vascular dysfunction: Key factors in the relationship between cerebrocardiovascular and psychological disorders. *Oxidative Medicine and Cellular Longevity*, *2019*, 4623109. 10.1155/2019/4623109 31814877 10.1155/2019/4623109 PMC6878772

[12] Jafari, M. J., Khosrowabadi, R., Khodakarim, S., & Mohammadian, F. (2019). The effect of noise exposure on cognitive performance and brain activity patterns. *Open Access Macedonian Journal of Medical Sciences*, *7*(17), 2924–2931. 10.3889/oamjms.2019.742 31844459 10.3889/oamjms.2019.742 PMC6901841

[13] Halperin, D. (2014). Environmental noise and sleep disturbances: A threat to health? *Sleep Science (Sao Paulo Brazil)*, *7*(4), 209–212. 10.1016/j.slsci.2014.11.003 26483931 10.1016/j.slsci.2014.11.003 PMC4608916

[14] Bisogno, A., Scarpa, A., Di Girolamo, S., De Luca, P., Cassandro, C., Viola, P., Ricciardiello, F., Greco, A., Vincentiis, M., Ralli, M., & Di Stadio, A. (2021). Hearing loss and cognitive impairment: Epidemiology, common pathophysiological findings, and treatment considerations. *Life (Basel Switzerland)*, *11*(10), 1102. 10.3390/life11101102 34685474 10.3390/life11101102 PMC8538578

[15] Baiduc, R. R., Sun, J. W., Berry, C. M., Anderson, M., & Vance, E. A. (2023). Relationship of cardiovascular disease risk and hearing loss in a clinical population. *Scientific Reports*, *13*(1), 1642. 10.1038/s41598-023-28599-9 36717643 10.1038/s41598-023-28599-9 PMC9886989

[16] Münzel, T., Gori, T., Babisch, W., & Basner, M. (2014). Cardiovascular effects of environmental noise exposure. *European Heart Journal*, *35*(13), 829–836. 10.1093/eurheartj/ehu030 24616334 10.1093/eurheartj/ehu030 PMC3971384

[17] European Environment Agency (2023, July 10). How does environmental noise pollution impact my health? European Union. https://www.eea.europa.eu/en/about/contact-us/faqs/how-does-environmental-noise-pollution-impact-my-health#:~:text=The%20EEA%20estimates%20that%20environmental,people%20suffer%20chronic%20sleep%20disturbance

[18] Beutel, M. E., Jünger, C., Klein, E. M., Wild, P., Lackner, K., Blettner, M., Binder, H., Michal, M., Wiltink, J., Brähler, E., & Münzel, T. (2016). Noise annoyance is associated with depression and anxiety in the general Population- the contribution of aircraft noise. *PloS One*, *11*(5), e0155357. 10.1371/journal.pone.0155357 27195894 10.1371/journal.pone.0155357 PMC4873188

[19] Liang, P., Li, J., Li, Z., Wei, J., Li, J., Zhang, S., Xu, S., Liu, Z., & Wang, J. (2024). Effect of low-frequency noise exposure on cognitive function: A systematic review and meta-analysis. *Bmc Public Health*, *24*, 125. 10.1186/s12889-023-17593-5 38195479 10.1186/s12889-023-17593-5 PMC10775542

[20] World Health Organization. (2022). *Compendium of WHO and other UN guidance on health and environment*. World Health Organization. https://iris.who.int/bitstream/handle/10665/352844/WHO-HEP-ECH-EHD-22.01-eng.pdf?sequence=1

[21] Centers for Disease Control and Prevention (2024a, April 12). What Causes Noise-Induced Hearing Loss. https://www.cdc.gov/nceh/hearing_loss/how_does_loud_noise_cause_hearing_loss.html

[22] Centers for Disease Control and Prevention (2020, December 14). Healthy People 2020. https://www.cdc.gov/nchs/healthy_people/hp2020.htm

[23] Hammer, M. S., Swinburn, T. K., & &Neitzel, R. L. (2014). Environmental noise pollution in the united States: Developing an effective public health response. *Environmental Health Perspectives*, *122*(2), 115–119. 10.1289/ehp.1307272 24311120 10.1289/ehp.1307272 PMC3915267

[24] The Lancet Regional Health-Europe. (2023). Noise pollution: More attention is needed. *The Lancet Regional Health Europe*, *24*, 100577. 10.1016/j.lanepe.2022.100577 36643665 10.1016/j.lanepe.2022.100577 PMC9832265

[25] Rosenthal, S. (2018). Motivations to seek science videos on YouTube: Free-choice learning in a connected society. *International Journal of Science Education Part B*, *8*(1), 22–39. 10.1080/21548455.2017.1371357

[26] Dixon, S. J. (2024). Most popular social networks worldwide as of January 2024, ranked by number of monthly active users(in millions). [Data set]. Statista. https://www.statista.com/statistics/272014/global-social-networks-ranked-by-number-of-users/

[27] Basch, C. H., & Basch, C. E. (2020). Emerging methods in health-related social media research: A case study of vaccine safety and Youtube. *Sage research methods cases: Medicine and health*. SAGE Publications, Ltd. 10.4135/9781529725575

[28] Nachar, N. (2008). The Mann-Whitney U: A test for assessing whether two independent samples come from the same distribution. *Tutorials in Quantitative Methods for Psychology*, *4*. 10.20982/tqmp.04.1.p013

[29] Mohamed, F., & Shoufan, A. (2024). Users’ experience with health-related content on YouTube: An exploratory study. *Bmc Public Health*, *24*(1), 86. 10.1186/s12889-023-17585-5 38172765 10.1186/s12889-023-17585-5 PMC10765842

[30] American Academy of Audiology (2020, February 12). A New Way to Prevent Noise-Induced Hearing Loss. https://www.audiology.org/a-new-way-to-prevent-noise-induced-hearing-loss/

[31] Academy of Audiology (2023, February 7). Another Kind of Pollution: How Human Noise Is Affecting Whales. https://www.audiology.org/another-kind-of-pollution-how-human-noise-is-affecting-whales/

